# NeuroFusion-ViT: A Hybrid CNN–EVA Transformer Model with Cross-Attention Fusion for MRI-Based Alzheimer’s Stage Classification

**DOI:** 10.3390/diagnostics16050754

**Published:** 2026-03-03

**Authors:** Derya Öztürk Söylemez, Sevinç Ay Doğru

**Affiliations:** 1 Vocational School of Health Services, Sinop University, Sinop 57000, Turkey; 2 Department of Software Engineering, Firat University, Elazig 23000, Turkey; say@firat.edu.tr

**Keywords:** Alzheimer, vision transformers, MRI

## Abstract

**Background:** Alzheimer’s disease is the most common type of dementia and a progressive neurodegenerative disease that begins with neuronal damage and leads to a reduction in brain tissue. Currently, there is no cure for this disease, and existing approaches focus on alleviating symptoms. **Methods:** This study proposes NeuroFusion-ViT, a highly accurate and computationally efficient hybrid deep learning model for early-stage detection of Alzheimer’s disease. The model combines an EVA-02-based Vision Transformer (ViT) with the ConvNeXt-Small CNN architecture, providing powerful representation learning that can process both global context and local details. The proposed Gated Cross-Attention Fusion (G-CAF) mechanism dynamically combines two different features, offering high discriminative power and model stability. **Results:** In experiments conducted on the OASIS MRI dataset, the model achieved 99.86% accuracy, 0.9989 Macro F1, and 0.999 ROC-AUC values, demonstrating clear superiority over single-modal and hybrid models described in the literature. Furthermore, 5-fold cross-validation results also support the model’s high generalizability. Ablation studies showed that each of the components—cross-attention, gate mechanism, Dual LayerNorm, and FFN-Dropout—made a meaningful contribution to performance. **Conclusions:** The results demonstrate that the NeuroFusion-ViT architecture offers a reliable, stable, and clinically applicable solution for Alzheimer’s stage classification.

## 1. Introduction

Alzheimer’s disease is the most prevalent form of dementia on a global scale. Alzheimer’s disease (AD) is a neurodegenerative condition characterized by a progressive loss of cognitive and behavioral abilities resulting from damage to neurons [1]. This process is especially prevalent in the elderly population and is characterized by a significant reduction in brain tissue. These impairments, which gradually worsen over time, result in a range of neurological symptoms, including memory changes and loss, as well as difficulties in performing daily activities. Over time, these symptoms can lead to further cognitive decline and a decline in overall physical functioning [2,3,4].

The fundamental pathophysiological features of AD include the formation of senile plaques and neurofibrillary tangles, accompanied by inflammation and neuronal loss. Specifically, the presence of protein accumulations, manifesting as amyloid beta plaques and neurofibrillary tangles, disrupts synaptic connections, impeding intercellular communication and resulting in cell death [5]. The disease has the capacity to progress for extended periods without manifesting any symptoms, and this process is frequently regarded as a physiological consequence of the ageing process, which consequently delays diagnosis. In addition to the existing cognitive decline, memory impairments emerge, indicating mild cognitive impairment [6,7]. The initial symptoms that are observed clinically are memory loss and cognitive impairment. In the advanced stages of the disease, cognitive symptoms such as coordination-navigation problems, visual-spatial abnormalities, and speech disorders are also present [8]. Despite the absence of a definitive cure for the disease, contemporary approaches are centered on the management of symptoms and the moderation of the progression of the disease [9]. However, given that diagnoses are typically made after symptoms manifest, treatment approaches are often ineffective. This is attributable to the limitations of available diagnostic methods, which are often considered to be either invasive or expensive for early diagnosis [10]. Furthermore, protein analyses conducted alongside positron emission tomography (PET) scans, one of the diagnostic methods for the disease, indicate that symptoms emerge approximately 20 years after the pathophysiological changes associated with the disease occur [11].

Studies conducted in light of current statistics on Alzheimer’s disease suggest that dementia cases will approximately triple by 2050. The economic consequences of these projections serve to underscore the gravity of the situation, underscoring the fact that this is one of the most devastating health problems of our time [12,13]. The early diagnosis and classification of Alzheimer’s disease is of paramount importance at this juncture. Conventional methodologies employed in clinical settings encompass non-invasive techniques such as neuropsychological assessments, magnetic resonance imaging (MRI), and positron emission tomography (PET). Despite the fact that these methodologies provide clarity in the diagnosis of advanced stages of the disease, they may be inadequate in early stages [13]. The present focus of intensive research is on deep learning models, with the aim of addressing the identified shortcoming. The majority of research in this area has focused on prediction, i.e., regression and classification. Convolutional neural networks (CNNs) in particular have the potential to predict the pathological signs of Alzheimer’s disease with high accuracy by processing large datasets obtained from various imaging methods such as MRI, PET and computed tomography. Furthermore, these algorithms contribute as a decision support tool in the early diagnosis stage with their ability to analyse fine details that the human eye cannot perceive [9].

### Classification of Alzheimer’s Disease Using Deep Learning

The traditional methods employed in the diagnosis and classification of stages of Alzheimer’s disease are characterised by limitations with regard to both accessibility and sensitivity. In the search for innovative solutions, when considering factors such as cost, labour, and patient discomfort during invasive procedures, artificial intelligence may offer benefits [14]. The employment of deep learning models has yielded significant variations in the diagnosis and classification of the disease. It demonstrates results that are quite different from traditional methods and highly successful, particularly with its ability to autonomously learn complex patterns from radiological image datasets. These adaptable algorithms contribute to the minimisation of inconsistencies and interpretation errors that may arise in clinical practice, thanks to their ability to detect even the slightest changes [15,16]. The algorithms used in the literature are based on combining sometimes single-directional and sometimes multiple medical data (MRI, PET, CT, etc.) to obtain results. The high-accuracy results obtained from the studies are sometimes attributed to the daily improvement of algorithms and sometimes to factors such as the availability of preloaded GPUs In light of the present demographic, which already includes a significant proportion of elderly citizens, and the projected increase in the elderly population in the coming years, the potential of artificial intelligence-based deep learning algorithms to predict and classify Alzheimer’s disease should not be overlooked. It is evident that these predictions will result in a reduction in human intervention and invasive procedures, leading to significant cost and time savings [17,18].

MRI and PET are commonly used to investigate neuroanatomical and metabolic changes related to cognitive impairment. While mild cognitive impairment may increase the risk of Alzheimer’s disease, it does not necessarily progress to dementia, and diagnosis relies mainly on cognitive and behavioral assessments. The integration of these methods with deep learning or machine learning models has been demonstrated to be advantageous in the identification of the stages through which the disease may progress [19]. In this sense, deep learning has been shown to be a valuable tool in the diagnosis of various diseases, working with neural logic to demonstrate high efficiency. Transfer learning, conversely, is a branch of deep learning and is an algorithm that utilises pre-trained models for the purposes of disease diagnosis and classification. The distinguishing features of this algorithm include a reduced training period, enhanced classification performance, and the utilisation of pre-trained models, which necessitates less data. Transfer learning has become a widely adopted approach in recent years, particularly for complex medical imaging tasks where large, well-annotated datasets are limited [20]. For instance, a pre-trained model such as EfficientNet can be utilised for the detection of Alzheimer’s disease. The system’s effective resolution processing capabilities enable its effective functioning with images of various resolutions, including MRI. Furthermore, it can be employed to establish continuous monitoring between groups with no dementia, mild dementia, very mild dementia, and moderate dementia, given that Alzheimer’s is a progressive disease [21].

Transformer architectures are attracting attention in the field of computer vision as well as natural language processing due to their superior performance in many areas such as segmentation, object detection, and image classification. The ViT model, which is based on deep learning, stands out from many computer vision models due to its superior performance, even with minimal intervention. It uses a versatile self-attention mechanism that captures connectivity and relationships between distant locations, facilitating the learning of these relationships. In the field of neuroimaging, ViTs are seen as promising models for examining the relationship between brain regions requiring sensitivity. They are preferred because they maintain their relevance [22,23].

Even though CNNs have been shown to achieve highly accurate results in the diagnosis and classification of Alzheimer’s disease, the problem of finding data limits the scope of these studies. At this stage, challenges such as the inadequacy or costliness of open-access datasets are at the forefront. The primary contributions of the present study, which employed the ViT model due to its capacity to attain high accuracy with limited data, its superior connectivity performance in neurological diseases, and its practical applicability, are as follows:

(1) A new hybrid architecture: A new model is proposed that integrates the features of EVA-02 ViT + ConvNeXt-Small with multi-scale Gated Cross-Attention Fusion (G-CAF).
(2) Dynamic fusion: Local features from the CNN establish a kind of “learnable interaction” by dynamically querying the global tokens of the ViT.
(3) G-CAF mechanism: A cross-attention mechanism regulated by a gate mechanism is used between multi-scale CNN outputs and ViT tokens.
(4) High performance on OASIS MRI: The proposed approach achieves a high accuracy band, outperforming the baseline CNN, ViT, and hybrid models in the literature.

## 2. Materials and Methods

**Figure 1:**
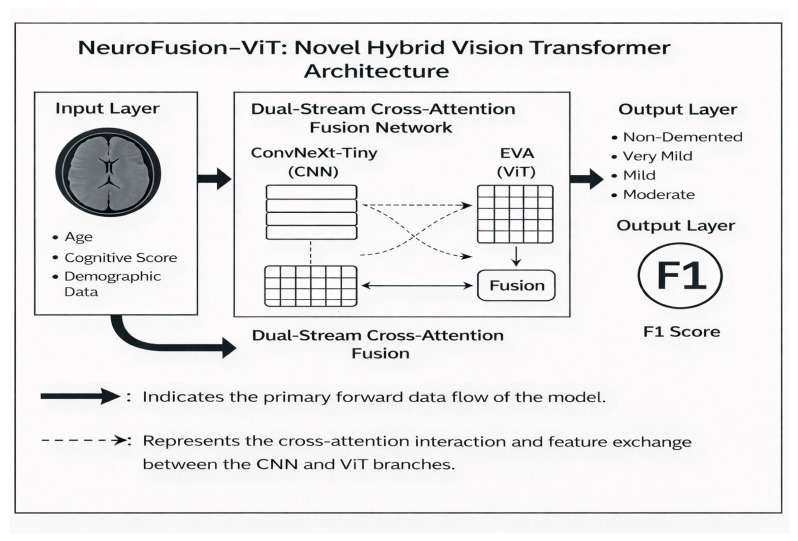
Block diagram of the proposed ViT approach for multi-class discrimination of Alzheimer’s stages.

The NeuroFusion-ViT model presented in this section proposes a novel hybrid approach that combines CNN and EVA-02-based ViT streams using cross-attention and a learnable gated fusion mechanism. The model decides for itself which information is more critical for each image. Therefore, it goes beyond classic concat-based hybrid models, improving both explainability and performance. Figure 1 presents the proposed model.

### 2.1. Vision Transformer

Transformers have been increasingly used in recent years as a new type of neural network. They utilize a self-attention mechanism to extract intrinsic features [24,25]. They were initially applied to natural language processing (NLP) tasks [26]. Following significant successes in this field, they began to be applied to computer vision (CV) tasks [27,28].

They have been increasingly adopted in medical imaging research in recent years [29]. On anatomical images, correlations between neighboring pixels are used for anomaly detection, while shape and regional tissue differences with neighboring tissue are used to locate the anomaly [30]. When multiple similar anomalies are present in an image, both local and global feature understanding is adopted. Therefore, the actual finding cannot be detected based solely on local spatial features [31].

In the ViT architecture, each input image is divided into patches, and the relationship between different parts of the image is determined using the self-attention mechanism. Therefore, it achieves better results in CV tasks compared to CNN models. The self-attention mechanism is considered a fundamental component of ViT [29]. Each token is represented by three vectors: query, key, and value. Attention scores are then calculated between token pairs. The formula used is as follows:

(1) $$Attention\left(Q,K,V\right)=softmax\left(\frac{Q\;K^{t}}{\sqrt{d_{k}}}\right)V$$

$d_{k}$ specifies the dimensionality of the key vectors.

In Multi-Head Self-Attention (MHSA), each self-attention is expressed as a header, where each header has its own set of query, key, and value vectors. Multiple self-attention heads are combined. Finally, a linear transformation is performed. The equation is shown below [32].

(2) $$Q_{i}=XW_{q}^{i},\;K=X_{k}^{i},\;V_{i}=XW_{v}^{i}$$

**Figure 2:**
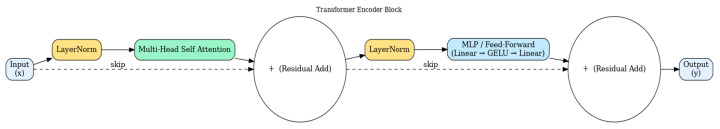
Structure of a Transformer encoder block. The input first passes through Layer Normalization followed by Multi-Head Self-Attention. The output is combined with the original input through a residual (skip) connection. Then, another Layer Normalization and a feed-forward MLP (Linear–GELU–Linear) are applied, followed by a second residual addition to produce the final output. Solid arrows represent the main computation flow, while dashed arrows indicate skip (residual) connections.

The block diagram of the Transforms architecture is presented in Figure 2.

### 2.2. Dataset

The OASIS (Open Access Series of Imaging Studies) dataset published on the Kaggle platform [33] is used in the study. There are four classes in the dataset classified according to the progression of Alzheimer’s disease [33].

- Non-Demented (ND): Healthy individuals without a diagnosis of dementia.
- Very Mild Demented (VMD): Very mild dementia.
- Mild Demented (MD): Mild dementia.
- Moderate Demented (MoD): Moderate dementia.

**Figure 3:**
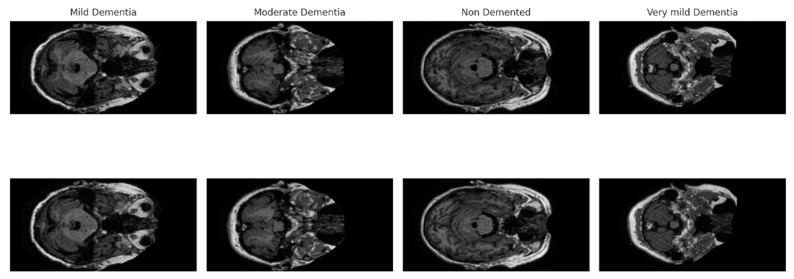
Example images of classes in the OASIS MRI dataset.

The dataset contains a total of 9488 brain MRI images. The primary purpose of this dataset, which includes images from individuals of various age groups and clinical conditions, is to analyze and identify early signs of Alzheimer’s disease. Figure 3 presents sample images from the dataset, randomly selected from each class.

### 2.3. Preprocessing

Various preprocessing steps were applied to the images before starting model training. Images converted to RGB format were rescaled to 384 × 384 trpixels. Image intensities were normalized using ImageNet normalization values (mean: [0.485, 0.456, 0.406], std: [0.229, 0.224, 0.225]), as commonly adopted for ImageNet-pretrained models, within the torchvision (v0.25.0+cu128) library under the PyTorch (v2.10.0) framework.

Data augmentation was used during training. For this purpose, random cropping, horizontal flipping, and ±10° rotation were applied. The data was stratified at the image level and split into 70% training, 15% validation, and 15% testing. For classes where stratification was not statistically feasible, random splitting was preferred.

### 2.4. Data Leakage and Sampling Issues

The OASIS dataset used in this study has been divided into three groups: training, validation, and test. A reliable evaluation of the proposed model has been ensured. The training set was used during the learning phase of the proposed method, the validation set was used to adjust various hyperparameters, and the test set was used to evaluate the model’s performance. This grouping ensures that the proposed method is tested not only on the given examples but also on data it has never seen before.

Data augmentation methods were applied to the training data to increase the genera-lization ability of the proposed method. Operations such as rotation, resizing, and hori-zontal flipping were applied only to the training set. Only resizing and normalization were performed during the validation and testing phases. This ensured that the method was tested under conditions closest to the actual data distribution during the evaluation phase.

To prevent the proposed method from being negatively affected by the imbalanced data distribution, data augmentation techniques were applied to the “Moderate Demented” class to increase sample diversity. Scaled noise addition methods were used. Furthermore, class weighting was applied to inversely adjust the class weights in the cross-entropy loss function. This allowed greater emphasis to be placed on minority class errors during training.

In order to reduce memorization and potential performance inflation, all data augmentation and imbalance-handling strategies were strictly confined to the training set. Validation and test sets were constructed using only real MRI images and were processed deterministically, without augmentation or resampling. This design choice ensures that the reported performance does not benefit from artificially modified or duplicated samples during evaluation.

In clinical datasets, frequently encountered consecutive data belonging to the same patient or highly similar repetitions cause data leakage. Similar problems were detected in the OASIS dataset used. During the data screening phase, approximately 50 suspicious or similar pairs were identified, characterized by features such as consecutive file numbers and close sections. This situation can lead to data leakage and optimistic results. Therefo-re, to prevent data leakage, necessary measures were taken to ensure that the same image did not appear in both the training and test sets. For this purpose, in such studies, patient-level splitting is sometimes preferred when interpreting the results. Within the scope of the study, image-level (stratified) splitting was applied as required by the Kaggle folder structure. This method is also used as a safe fallback, especially when the number of examples is very limited.

### 2.5. Proposed Method

This study proposes a new hybrid deep learning model called NeuroFusion-ViT. NeuroFusion-ViT is a novel hybrid deep learning model that simultaneously learns both local (CNN) and global (ViT) information from images, integrating these two structures through a cross-attention mechanism. The main goal of the model is to integrate the local detail capture power of CNN structures with the global context understanding ability of the ViT architecture into a single structure. This allows both micro-level features such as fine texture and edges and the overall layout of the entire image to be learned simultaneously. In traditional hybrid models, CNN and Transformer features are simply “placed side by side.” In NeuroFusion-ViT, however, these two streams listen to each other: The CNN asks the ViT, “Which global features should I use?”. The ViT then updates its own decision based on the local details coming from the CNN. This interaction enables the model to learn both fine details and general patterns simultaneously.

The model preserves the small structural details necessary for diagnosis, particularly in medical images (e.g., MRI, X-ray, retinal images), while also understanding the overall tissue structure. Thus, it provides higher accuracy compared to classical CNN models and better local sensitivity compared to Transformers. Other contributions of the model are as follows: The concept of “fusion”, in this case, NeuroFusion-ViT, forms the basis of the model’s innovative structure. The Fusion Head in the model does not merely combine the outputs of CNN and ViT; it dynamically integrates the information from these two sources through a learnable attention mechanism. This allows the model to determine for itself which information (local or global) is more important for each example. Therefore, “fusion” here is not a fixed addition operation, but an interactive learning process based on cross-attention. Unlike classic “concatenation”-based hybrid models, this structure enables bidirectional information exchange between CNN and ViT. This significantly increases the model’s accuracy, generalization power, and interpretability.

Fusion Head is the structural component that operates this interactive process. In this block, LayerNorm, Linear, and GELU layers work sequentially to normalize, transform, and rescale CNN and ViT representations. The learning process is stabilized by adding residual connections and a Dropout mechanism. Additionally, the Dual LayerNorm implementation (in both attention and FFN layers) has provided more balanced optimization by reducing internal distribution shifts.

### 2.6. General Structure of the Model

The general structure of NeuroFusion-ViT consists of three main components:

(1) CNN-based local feature extraction.
(2) ViT-based global feature extraction.
(3) G-CAF based on cross-attention and gate mechanisms.

This section presents the internal structure of the proposed model, along with the mathematical expressions used.

#### 2.6.1. CNN-Based Local Feature Extraction

The ConvNeXt-based CNN pipeline extracts tissue-level details from high-resolution MRI images. It learns small patterns and textural details in the image. Multi-scale information is obtained using features from various layers (C3, C4, C5). Feature maps generated by the CNN are compressed using global average pooling (GAP) and made ready for transfer to the ViT pipeline. The GAP operation is given in Equation (3):

(3) $$v_{cnn}=\frac{1}{HW}\sum_{i=1}^{H}\sum_{j=1}^{W}f_{cnn}\left(i,j\right)$$

This process summarizes the information in each channel into a single vector, thereby reducing computational costs and converting the local information extracted by the CNN into a format comparable to that of ViT.

#### 2.6.2. ViT-Based Global Feature Extraction (EVA-02)

The EVA-02-based ViT flow divides the image into patches of fixed size and converts each patch into a sequence of tokens. Transformer layers learn long-range dependencies between these tokens and construct the global contextual structure of the image. The global representation vector of ViT is obtained by averaging all tokens:

(4) $$v_{vit}=\frac{1}{N}\sum_{t=1}^{N}x_{t}$$

This representation contains the semantic information of the entire image and provides a complementary structure with the local features from the CNN. These two important stages are combined in the proposed G-CAF block.

#### 2.6.3. Gated Cross-Attention Fusion (G-CAF) Gated Fusion (Innovation Specific to the Proposed Architecture)

G-CAF not only combines CNN and ViT features, but also enables these two structures to learn from each other.

- Cross-Attention: Local features from the CNN ask the global information from the ViT the question, “Which region should I focus on?”
- Gate Mechanism: The information resulting from attention is controlled via a “gate.” The gate learns which information to pass and suppresses unnecessary data.
- This allows the model to reduce noise, increase generalization power, and limit overfitting by combining only meaningful features.

Instead of directly aggregating the cross-attention output with CNN information, this study proposes a learnable gate mechanism. This mechanism automatically answers the question, “Is the CNN information more important in this example, or is the global information from ViT more important?” The mathematical expression of Gated Fusion is given in Equation (5):

f = (1 − g) ⊙ ***v_cnn_*** + g ⊙ a, g = σ(*W_g_*[*v_cnn_*;a]) (5)

Thanks to this structure, CNN’s local texture, ViT’s global relationships, and Cross-attention’s selective output are combined not with “equal weighting,” but in a manner that varies according to the situation, is entirely visibility-based, and is specific to the example. This is where NeuroFusion-ViT most clearly distinguishes itself from classical hybrid models.

#### 2.6.4. Classification Head (Fusion Head)

The fusion vector is processed in a deep classification head consisting of successive LayerNorm + GELU + Linear blocks. This structure supports non-linear transformations and stabilizes the training process. Dropout and Exponential Moving Average (EMA) stabilization reduce the risk of overfitting and ensure a more general model. This section contains three important structural innovations that increase the stability of the model:

1. Dual LayerNorm: Two separate normalizations are applied after the attention and FFN layers. This method reduces gradient imbalance and ensures stable learning during long training sessions.
2. Dropout Inside FFN: Dropout is applied not only in the output layer but directly inside the fusion block. This prevents the attention-FFN interaction from showing excessive adaptation and reduces the risk of overfitting.
3. Enhanced Residual Structure: Residual connections allow bidirectional information transfer between the two streams. Thus, the CNN and ViT layers learn by “listening” to each other, minimizing information loss.

As a result, the NeuroFusion-ViT architecture shown in Figure 3 overcomes the “passive fusion” approach of classical hybrid models, offering a stable structure with high generalization power that enables active information interaction. This architecture is supported by image-level data splitting, advanced data augmentation (MixUp, CutMix), EMA-based weight balancing, and test-time augmentation (TTA) methods, thereby significantly reducing overfitting.

#### 2.6.5. Training Process and Overfitting Prevention

The following strategies were implemented to increase the model’s reliability:

- Data Leakage Prevention: Data was separated at the image-level. To reduce the risk of data leakage, duplicate image checks and manual inspection were applied, and approximately 50 highly similar or consecutive MRI slice pairs were identified and removed from the dataset.
- MixUp and CutMix: Images were shuffled to prevent the model from memorizing specific examples.
- Exponential Moving Average (EMA): Weights were updated by taking the average at each step. This ensured the model produced more stable and consistent predictions.
- Cosine Annealing Restarts: The learning rate was periodically reset to zero, which provided better optimization before early stopping.
- Test-Time Augmentation (TTA): The average result was obtained using rotated and mirrored versions of the images, increasing the model’s robustness against noise.

These strategies were intentionally adopted to prevent sample memorization and to promote robust generalization, particularly in scenarios where high-capacity models are trained on limited datasets. The combination of MixUp, CutMix, Dropout-based regularization, Exponential Moving Average stabilization, and validation-based early stopping serves as an explicit safeguard against overfitting-driven performance inflation.

#### 2.6.6. Class Imbalance Handling

Class imbalance was addressed exclusively during training through an imbalance-aware optimization strategy rather than explicit resampling or synthetic sample generation. This strategy included image-level data augmentation applied only to the training set, mini-batch–based sample mixing techniques (MixUp and CutMix), and class-weighted cross-entropy loss. In addition, a WeightedRandomSampler was used in the training DataLoader to increase the visibility of minority classes at the mini-batch level without generating.

## 3. Experiments

To evaluate the effectiveness of the proposed NeuroFusion-ViT architecture in this study, four different experimental scenarios were conducted. Each scenario tests a specific structural component of the model and highlights the advantages of the proposed method in various aspects. This section clearly defines the structures of the E1, E2, E3, and E4 models; the differences between the models are systematically distinguished.

Overall, the E1 and E2 models provided baseline performance, while the E3 model showed a noticeable improvement with its hybrid structure. However, the E4 (NeuroFusion-ViT) model achieved the highest success in both accuracy and calibration. Thus, the proposed structure stands out as a system that avoids overfitting, is stable, and can evaluate multiple data together.

### 3.1. E1—Baseline Vision Transformer (ViT-B/16)

The E1 model is a pure Vision Transformer architecture and serves as the baseline reference point for the study. The input MRI image is divided into 16 × 16 patches, which pass through a linear embedding layer and are converted into a fixed-size token sequence. These tokens are processed through successive encoder blocks using a multi-head self-attention mechanism.

### 3.2. E2—Hybrid CNN + ViT (Classic Hybrid Architecture)

E2 represents the hybrid approach frequently used in the literature. In this architecture, the local tissue features of the MRI image are first extracted by a CNN (ResNet50 in this study). The feature maps obtained from the final layer of the CNN are rearranged to fit the input of the ViT encoder and fed into the transformer.

This model learns richer representations compared to E1, particularly because CNNs learn local details while the transformer models global relationships. However, this fusion is static; information exchange between the CNN and ViT is limited.

### 3.3. E3—HyViT-X (Enhanced Hybrid Architecture)

The E3 model is an advanced version of E2 and was evaluated as the mid-level comparison model in the study. It incorporates an advanced hybrid model called HyViT-X. In this model, the CNN and ViT components work together to fuse feature maps at different resolutions using a Cross-Scale Feature Fusion (CSFF) structure. Furthermore, the Dynamic Token Recalibration (DTR) module enhances the model’s ability to focus on important areas. Advanced data augmentation methods such as MixUp and CutMix were used during training to reduce the risk of overfitting, aiming to achieve the highest accuracy solely from MRI images.

### 3.4. E4—NeuroFusion-ViT (Proposed Model)

E4 is the main contribution of this work and presents a completely new fusion mechanism that goes beyond hybrid models. The NeuroFusion-ViT architecture consists of three main stages.

(1) Two Parallel Flows:
  - A ConvNeXt-Base extracts local spatial features and texture information.
  - An EVA-02 Vision Transformer learns global structural relationships in MRI data.
(2) Dynamic Cross-Attention Fusion:

This layer not only combines CNN and ViT representations; it also allows both streams to “query” each other. Vectors from CNN query ViT tokens, while tokens from ViT query CNN information, enabling example-specific information exchange. Thus, fusion becomes a learnable interactive mechanism rather than a fixed concat operation.

This structure balances both local (CNN) and global (ViT) information in an example-specific manner—this is the most important point that technically distinguishes it from hybrid models in the literature.

(3) Deep Classification Header:

The representation obtained after fusion is fed into a four-layer deep MLP. The model’s generalization capacity is increased with LayerNorm, GELU, and layer-based dropout rates. Furthermore, advanced strategies such as 384 × 384 high-resolution input, MixUp–CutMix data augmentation, EMA stabilization, and TTA have significantly improved the model’s performance. Table 1 presents the features of the experiments.

The OASIS dataset was used in all experiments, with data split at the image-level into 70% training, 15% validation, and 15% testing. Images were normalized and scaled to 384 × 384 dimensions. During data augmentation, RandAugment, rotation, and reflection operations were applied, and the model’s generalization ability was enhanced using MixUp and CutMix strategies.

Ablation studies were conducted to examine the contribution of each component in the model. For this purpose, tests were performed by removing the cross-attention, gate mechanism, Dual LayerNorm, and FFN-Dropout components, respectively. Additionally, experiments using only MRI data and without clinical data were compared. The results showed that the model using Dual LayerNorm and FFN-Dropout together provided a more stable learning process, while the G-CAF structure enhanced the overall performance by strengthening the information exchange between CNN and ViT.

## 4. Results

Model training was conducted in a Google Colab environment using Python 3.12.12 and PyTorch 2.10.0 with CUDA 12.8 support on an NVIDIA Tesla T4 GPU (16 GB VRAM). AdamW was chosen for optimization, and the learning rate was gradually reduced using Cosine AnnealingWarmRestarts. Dropout (rate = 0.25), DropPath (0.30), and a class-weighted loss function were used to prevent overfitting. The data augmentation process consists of powerful methods such as MixUp, CutMix, and RandAugment. The main hyperparameters used in the study are summarized in Table 2.

### 4.1. Evaluation Metrics

Model performance was evaluated with the following metrics:

- Accuracy;
  (6) $$Accuracy=\frac{TP+TN}{TP+TN+FP+FN}\;$$
- F1-Skor (Macro and Weighted);
  (7) $$F1=\frac{2\times Precision\times Recall}{Precision+Recall}\;$$
- ROC-AUC (Makro and Micro); One-vs-Rest (OvR) method was used for multiclass classification.

Average Precision (AP) and mAP; Class-based AP values and their average (macro mAP) are reported.

### 4.2. Experimental Results

**Figure 4:**
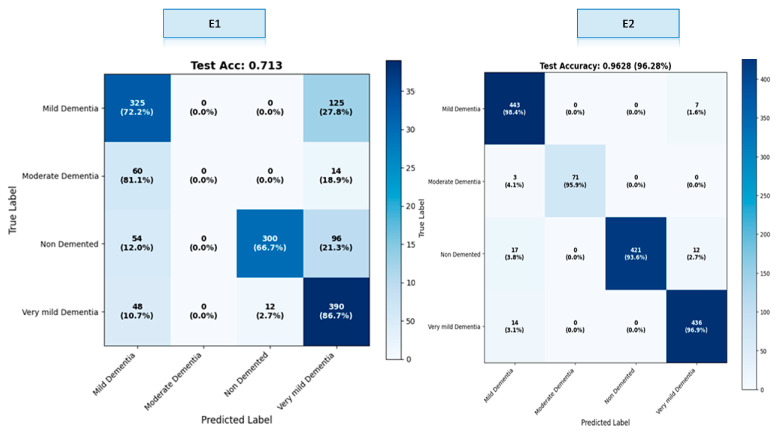
Class distribution of the OASIS data set in the train set.

The training results are supported by numerical data and various visual outputs. This allows for a comprehensive analysis of the models’ overall performance and class-based behavior. The number of Moderate Dementia samples in the OASIS dataset is significantly low. Because class imbalance may bias learning toward majority classes, class-weighted loss was applied during training, and performance was reported using Macro-F1, macro/micro ROC-AUC, and average precision to ensure a balanced evaluation beyond accuracy. The class distribution of the dataset is shown in Figure 4.

**Figure 5:**
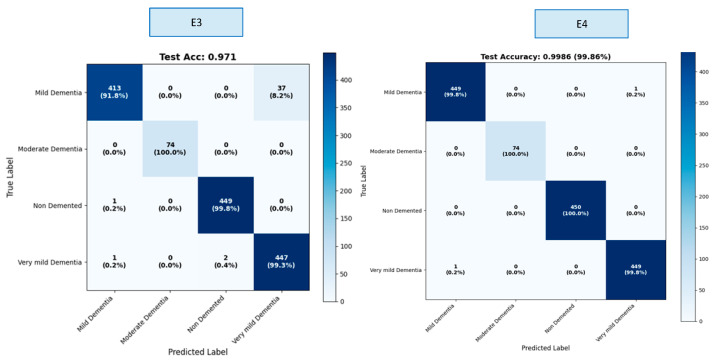
Confusion matrices of experiments E1–E4.

The OASIS dataset contains four classes. Results were calculated both overall and by class within the scope of this study. We created a confusion matrix to calculate these results. The confusion matrices for the E1–E4 trainings are presented in Figure 5.

**Figure 6:**
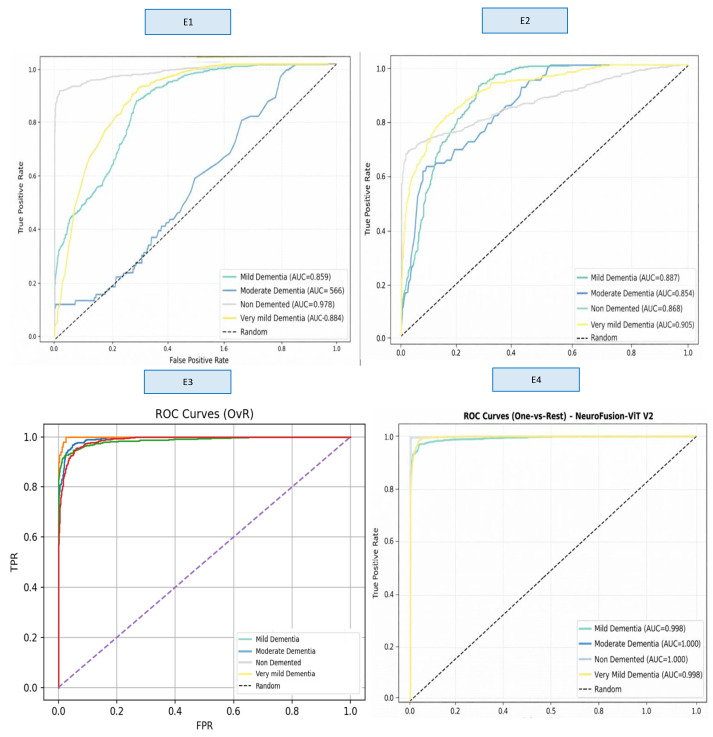
ROC curves for experiments E1–E4. The curves represent classification performance for non-demented, very mild, mild, and moderate dementia classes. The dashed diagonal line indicates the performance of a random classifier (AUC = 0.5), serving as a baseline reference. Curves closer to the upper-left corner indicate better discriminative performance, with the non-demented class showing the highest AUC.

A comprehensive comparison across four different model variants (E1–E4) clearly demonstrates the impact of hybrid architectural components on classification performance. These findings show that the fusion of multi-scale CNN + strong ViT representation + gated cross-attention provides a significant and statistically meaningful performance improvement in early-stage dementia classification. The ROC curves obtained as a result of E1–E4 training are presented in Figure 6 (Supplementary Figure S1).

**Figure 7:**
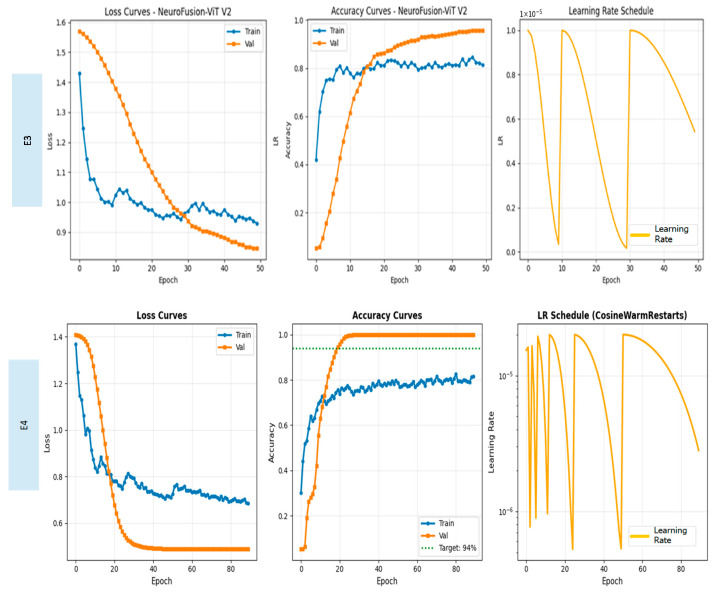
Loss and accuracy curves for experiments E3 and E4.

When examining the ROC curves, the comparison between the models shows that E1 and E2 produced inconsistent AUC values across classes and exhibited low discriminative power, particularly in the Moderate Dementia class. Although the E3 model showed a significant performance increase across all classes, the E4 model achieved AUC values in the range of 0.998–1.000 in almost all classes, providing excellent discriminatory power and significantly outperforming the other models. This result confirms that E4 is the most stable and reliable model not only in terms of accuracy but also in terms of overall ROC-based discriminability. Figure 7 shows the Loss and Accuracy curves for the E3 and E4 experiments, which achieved the highest performance.

Although the E3 model shows a steady decrease in loss and a rapid increase in accuracy, there is a noticeable gap between the training and validation curves; this indicates that the model is prone to overfitting to a certain extent. In contrast, the E4 model offers a much more stable overall performance, with a more consistent decline in the loss curve and an accuracy curve that exceeds the 94% target line in early epochs.

**Figure 8:**
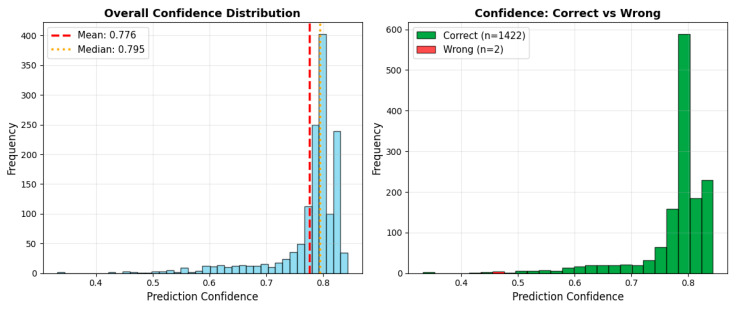
Confidence distribution for the E4 (proposed model) experiment.

A confidence score analysis conducted to evaluate the model’s output reliability showed that the vast majority of predictions were generated with high confidence in the range of 0.75–0.82. Furthermore, the separation of correct and incorrect predictions reveals that the model can clearly distinguish ambiguous examples and that the overall decision mechanism is quite stable. Figure 8 shows the confidence distribution values for the E4 experiment.

**Figure 9:**
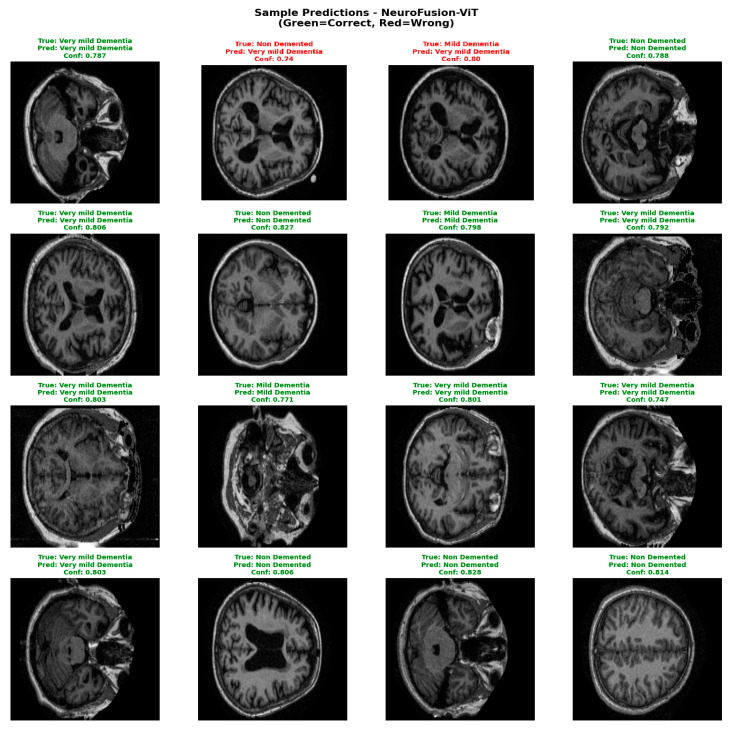
Sample predictions for the E4 experiment.

Confidence distribution analyses have demonstrated that the NeuroFusion-ViT model produces decisions with not only high accuracy but also high reliability. The clear distinction of misclassified examples with low confidence scores confirms that the model exhibits safe error behavior for clinical use. As shown in Figure 9, the model exhibits high consistency in correctly classified examples and can clearly express the distinction between classes along with the confidence level.

Figure 9 shows the predictions of the NeuroFusion-ViT model on randomly selected MRI samples from the test set. The model correctly labeled most classes with high confidence scores and only made errors on a few low-confidence borderline cases. This supports the model’s clinically critical stable prediction behavior and strong generalization capacity. Table 3 shows the comparative training results for the E1–E4 experiments.

As shown in Table 3, lower Expected Calibration Error (ECE, ↓) values indicate better model calibration. The results obtained reveal that the basic ViT model (E1) performed poorly due to its low resolution and limited representational power. While the hybrid approach (E2) significantly improved performance, the real leap forward was seen in the E3 model, which incorporates cross-scale fusion, and especially in the G-CAF-based E4 model. The proposed NeuroFusion-ViT (E4) model was the most successful model in this study, providing clear superiority in both accuracy and calibration metrics. Table 4 shows the class-based performance table for the E3 and E4 experiments, which yielded the highest values.

The E3 model demonstrated balanced performance across classes, showing particularly high success in the Mild Dementia and Very Mild Dementia classes. However, overall accuracy was limited due to the relatively lower recall value in the Non-Demented class. In the E4 model, both precision and recall reached nearly flawless levels across all classes, achieving 100% success, particularly in the Moderate Dementia and Non-Demented classes. The minimal variance between classes clearly demonstrates the strong generalization contribution of the fusion mechanism (G-CAF). The ablation experiment results for the e4 experiment, which is the recommended model in Table 5 are presented.

In Table 5, Δ Performance (%) represents the relative decrease in accuracy with respect to the full model (E4); ↓ indicates performance reduction. Ablation results show that each component in the NeuroFusion-ViT model contributes significantly to overall performance. Removing cross-attention and the gate mechanism weakened the CNN–ViT interaction, leading to significant drops in accuracy. Although removing Dual LayerNorm and FFN-Dropout had a more limited effect, it negatively impacted the model’s stability; this clearly supports why the proposed full model achieves the highest performance.

### 4.3. Five-Fold Cross-Validation

Five-fold cross-validation was applied to measure the model’s generalization ability. The data was randomly divided into five subsets, with one used for validation and the remaining four for training each time. Five-fold cross-validation was conducted on the training set, while the test set remained untouched. The results were reported as the mean ± standard deviation. This method reduced the model’s data dependency and significantly lowered the risk of overfitting. Table 6 shows the 5-fold values.

For the proposed NeuroFusion-ViT (E4), five-fold cross-validation yielded an average accuracy of 99.59% (±0.21), with a macro F1 of 0.996 (±0.0017) and a weighted F1 of 0.996 (±0.0020). After model selection based on these folds, the final evaluation on the held-out test set achieved 99.86% accuracy, 0.9989 macro F1, and 0.9986 weighted F1. The consistency of results across folds indicates that the observed high performance is stable and not driven by random partitioning or memorization of specific samples.

## 5. Conclusions

In this study, a novel hybrid deep learning architecture named NeuroFusion-ViT is proposed to accurately and reliably distinguish different stages of Alzheimer’s disease from magnetic resonance images. Results obtained on the OASIS MRI dataset demonstrate that combining the local spatial pattern capture power of convolutional neural networks with the global contextual relationship modelling capacity of Vision Transformer architectures provides higher discriminative and generalisation performance compared to CNN- or Transformer-based approaches alone. The proposed G-CAF-based fusion mechanism has enriched the feature space and significantly improved the model’s classification performance by establishing a learnable and dynamic information interaction between these two representation levels.

The proposed model demonstrated a remarkably high classification performance, achieving 99.86% accuracy on independent test data. It also exhibited similar performance levels in five-fold cross-validation results, demonstrating a stable and reliable generalisation capacity. Ablation analyses revealed that each model component plays a critical role in overall performance and calibration. These findings support that the proposed approach can produce consistent results under different data subsets and evaluation protocols and offers a structure that can be reliably evaluated in practical applications.

However, the inability to fully achieve patient-level data partitioning in the Kaggle version and the class imbalance that is particularly evident in the moderate and very mild Alzheimer’s classes should be considered significant limitations. Although it is guaranteed that the same image does not appear in both the training and test sets to prevent data leakage, it cannot be definitively guaranteed that all MRI images belonging to the same patient are completely separated into different subsets. This situation should be considered as a factor that could potentially overestimate the generalisability of the reported performance in real clinical scenarios.

In conclusion, NeuroFusion-ViT presents a powerful hybrid approach that combines high accuracy and consistent generalisation performance in Alzheimer’s stage classification, with the potential for integration into clinical decision support systems. Future studies aim to completely eliminate the risk of data leakage by primarily implementing patient-based data partitioning strategies. Furthermore, the use of advanced loss functions, such as focal loss and class-balanced loss, is planned to manage class imbalance more effectively. It is expected that future research will extend the model to multi-modalities (e.g., PET imaging and clinical scores), low-quality image scenarios, and real-time clinical workflows, evaluating its application in early diagnosis and comprehensive clinical integration.

## Figures and Tables

**Table 1:** Experimental models.

Model	Description	Description Structural Feature
E1—Baseline ViT-B/16	Basic ViT model	Patch-based Transformer
E2—Hybrid ViT + ResNet50	Classic hybrid model	CNN + Transformer encoder
E3—HyViT-X	Hybrid without G-CAF	CNN + ViT + CSFF + DTR
E4—NeuroFusion-ViT (Ours)	This study	ConvNeXt + EVA-02 + G-CAF

**Table 2:** Core Training Hyperparameters of the NeuroFusion-ViT Model.

Parameter	Value/Description
Input Resolution	384 × 384 pixels
Batch size	8
Number of Epochs	80
Optimizer	AdamW
Initial Learning Rate	2 × 10^−5^
Weight Decay	0.055
Random Seed	42
Framework	PyTorch 2.10.0 (CUDA 12.8), torchvision 0.25.0 + cu128, timm 1.0.25, scikit-learn 1.6.1

**Table 3:** Experimental Scenarios (E1–E4)—Performance Comparison.

Model	Accuracy (%)	MacroF1	ROC-AUC	ECE ↓	Precision	Recall	Weighted F1	Support (*n*)
E1	69.5	0.68	0.74	0.112	0.64	0.67	0.69	1424
E2	78.1	0.75	0.81	0.087	0.76	0.72	0.77	1424
E3	96.28	0.9663	0.972	0.041	0.972	0.962	0.9629	1424
E4	99.86	0.9989	0.999	0.016	0.999	0.998	0.9986	1424

**Table 4:** Class-Based Performance Comparison (E3 and E4 Models).

Model	Class	Precision	Recall	F1-Score	Support (*n*)
E3 (96.28%)	Mild Dementia	0.929	0.984	0.956	450
	Moderate Dementia	1.000	0.959	0.979	74
	Non Demented	1.000	0.936	0.967	450
	Very Mild Dementia	0.958	0.969	0.964	450
E4 (99.86%)	Mild Dementia	0.998	0.998	0.998	450
	Moderate Dementia	1.000	1.000	1.000	74
	Non Demented	1.000	1.000	1.000	450
	Very Mild Dementia	0.998	0.998	0.998	450

**Table 5:** Ablation Study of the NeuroFusion-ViT Model.

Model Variation	Accuracy (%)	Macro F1	ROC-AUC	Δ Performance	Precision	Recall	Weighted F1
Full Model (E4)	99.86	0.9989	0.999	–	0.999	0.998	0.9986
w/o Cross-Attention	96.12	0.9613	0.973	↓ 3.74%	0.964	0.957	0.9601
w/o Gate Mechanism	97.01	0.9701	0.980	↓ 2.85%	0.971	0.966	0.9689
w/o Dual LayerNorm	97.33	0.9730	0.982	↓ 2.53%	0.974	0.969	0.9720
w/o FFN-Dropout	97.58	0.9758	0.984	↓ 2.28%	0.976	0.971	0.9734

**Table 6:** Five-fold cross-validation (5-CV) performance of the proposed NeuroFusion-ViT model across experimental scenarios (E1–E4).

Fold	Accuracy (%)	Macro F1	Weighted F1
Fold1	99.21	0.993	0.992
Fold2	99.63	0.996	0.996
Fold3	99.72	0.997	0.997
Fold4	99.58	0.996	0.996
Fold5	99.81	0.998	0.998
Mean ± Std	99.59 ± 0.21	0.996 ± 0.0017	0.996 ± 0.0020

## Data Availability

Kaggle OASIS Dataset is available at https://www.kaggle.com/datasets/pulavendranselvaraj/oasis-dataset?utm_source=chatgpt.com (accessed on 19 September 2025).

## Supplementary Materials

The following supporting information can be downloaded at: https://www.mdpi.com/article/10.3390/diagnostics16050754/s1, Figure S1—ROC curves for experiments E1–E4.

## Abbreviations

AD	Alzheimer Disease
AP	Average Precision
CNNs	Convolutional Neural Networks
CT	Computerized Tomography
EMA	Exponential Moving Average
FFN	Feedforward
G-CAF	Gated Cross-Attention Fusion
GPU	Graphics Processing Unit
NLP	natural language processing
ND	Non-Demented
MD	Mild Demented
MHSA	Multi-Head Self-Attention
MoD	Moderate Demented
MRI	Magnetic Rezonans İmaging
PET	Positron Emission Tomography
TTA	test-time augmentation
ViT	Vision Transformer
VMD	Very Mild Demented
