# Supplementary material for: NeuroFusion-ViT: A Hybrid CNN–EVA Transformer Model with Cross-Attention Fusion for MRI-Based Alzheimer’s Stage Classification
Source: Diagnostics (Basel). 2026 Mar 3;16(5):754. doi: 10.3390/diagnostics16050754 (PMC12984189; doi:10.3390/diagnostics16050754)
Supplement: Supplementary file 1 [file diagnostics-16-00754-s001.zip › diagnostics-4055527-supplementary.pdf]

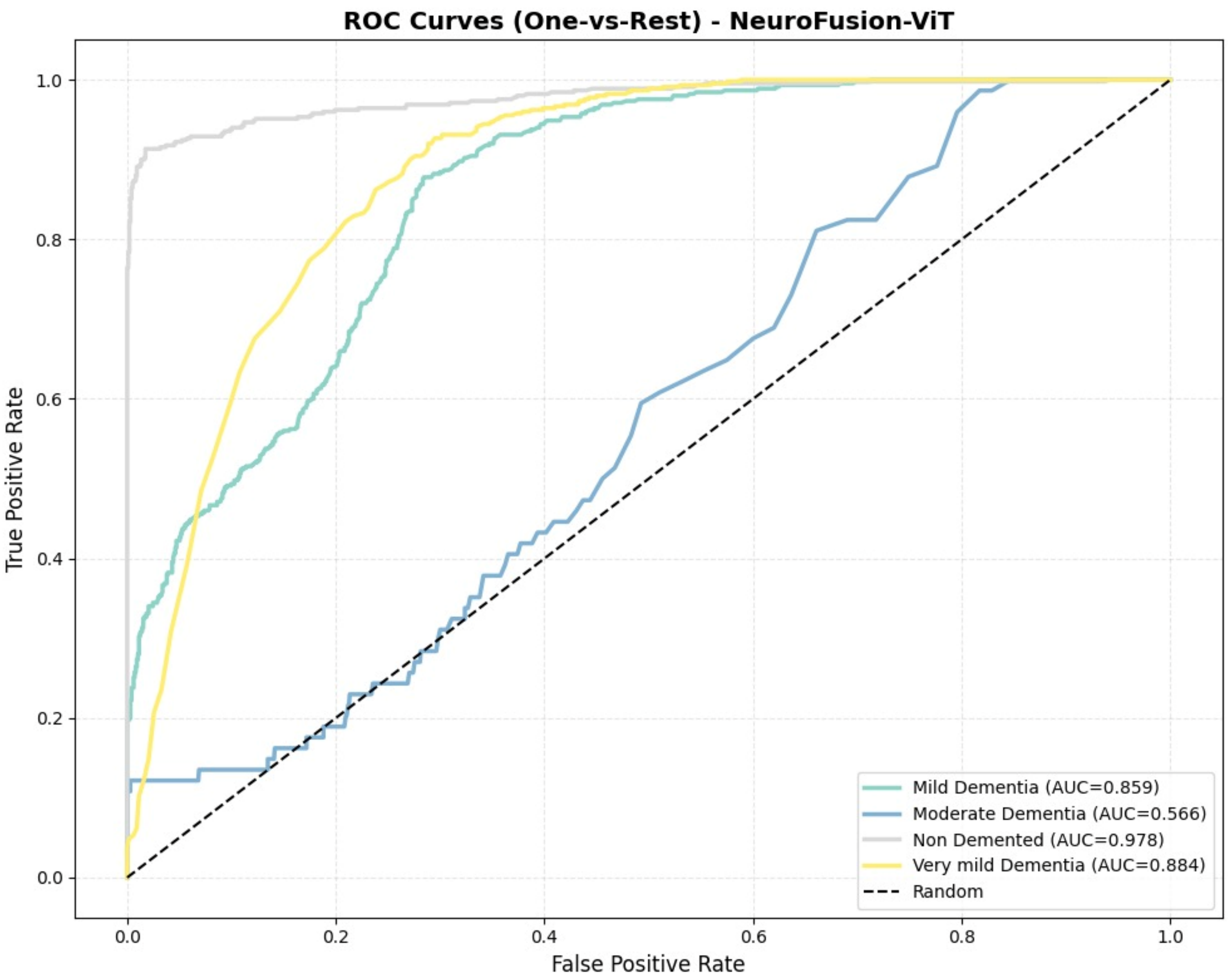
ROC Curves (One-vs-Rest) - NeuroFusion-ViT
True Positive Rate
False Positive Rate
0.0
0.2
0.4
0.6
0.8
1.0
Mild Dementia (AUC=0.859)
Moderate Dementia (AUC=0.566)
Non Demented (AUC=0.978)
Very mild Dementia (AUC=0.884)
Random

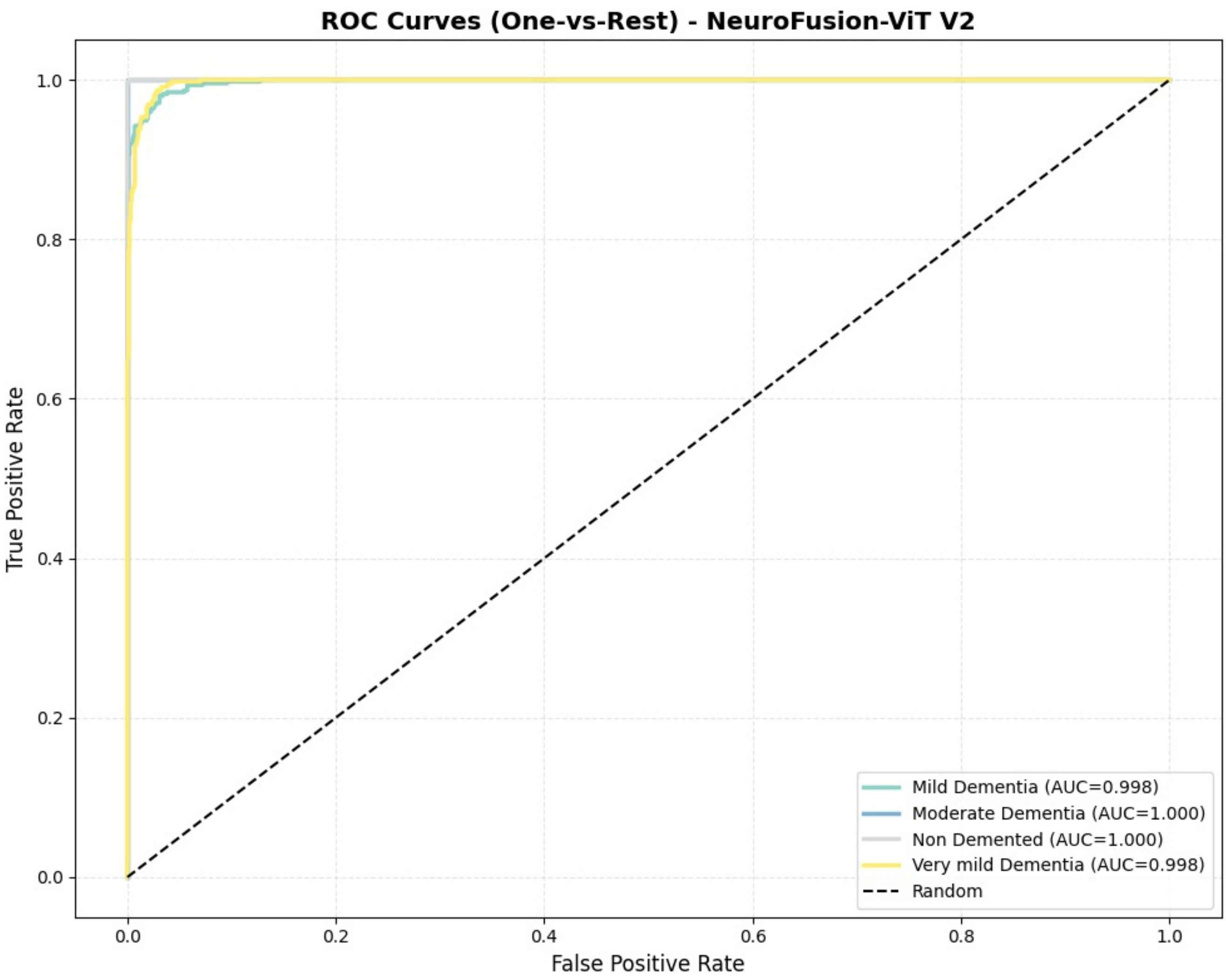
ROC Curves (One-vs-Rest) - NeuroFusion-ViT V2
True Positive Rate
False Positive Rate
0.0
0.2
0.4
0.6
0.8
1.0
Mild Dementia (AUC=0.998)
Moderate Dementia (AUC=1.000)
Non Demented (AUC=1.000)
Very mild Dementia (AUC=0.998)
Random

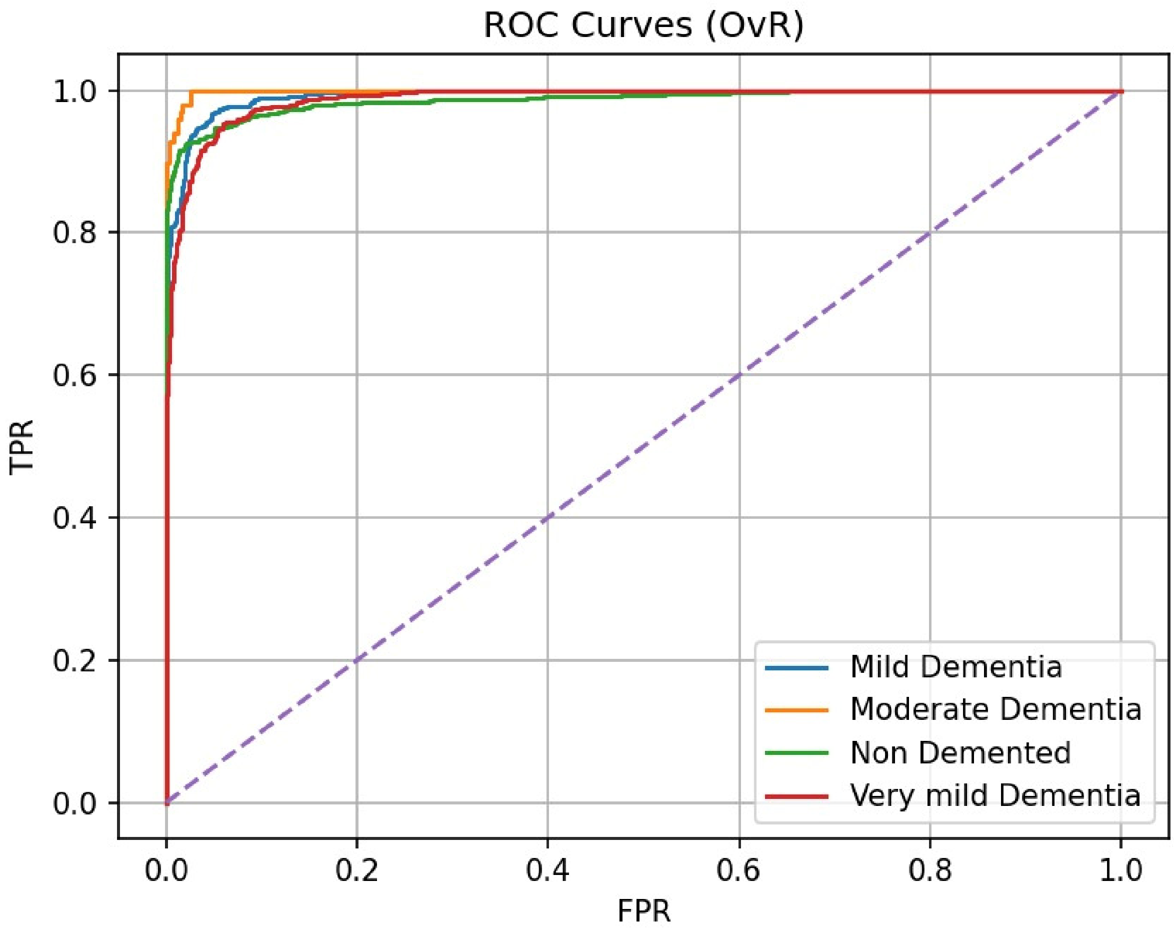
ROC Curves (OvR)
TPR
FPR
0.0
0.2
0.4
0.6
0.8
1.0
Mild Dementia
Moderate Dementia
Non Demented
Very mild Dementia

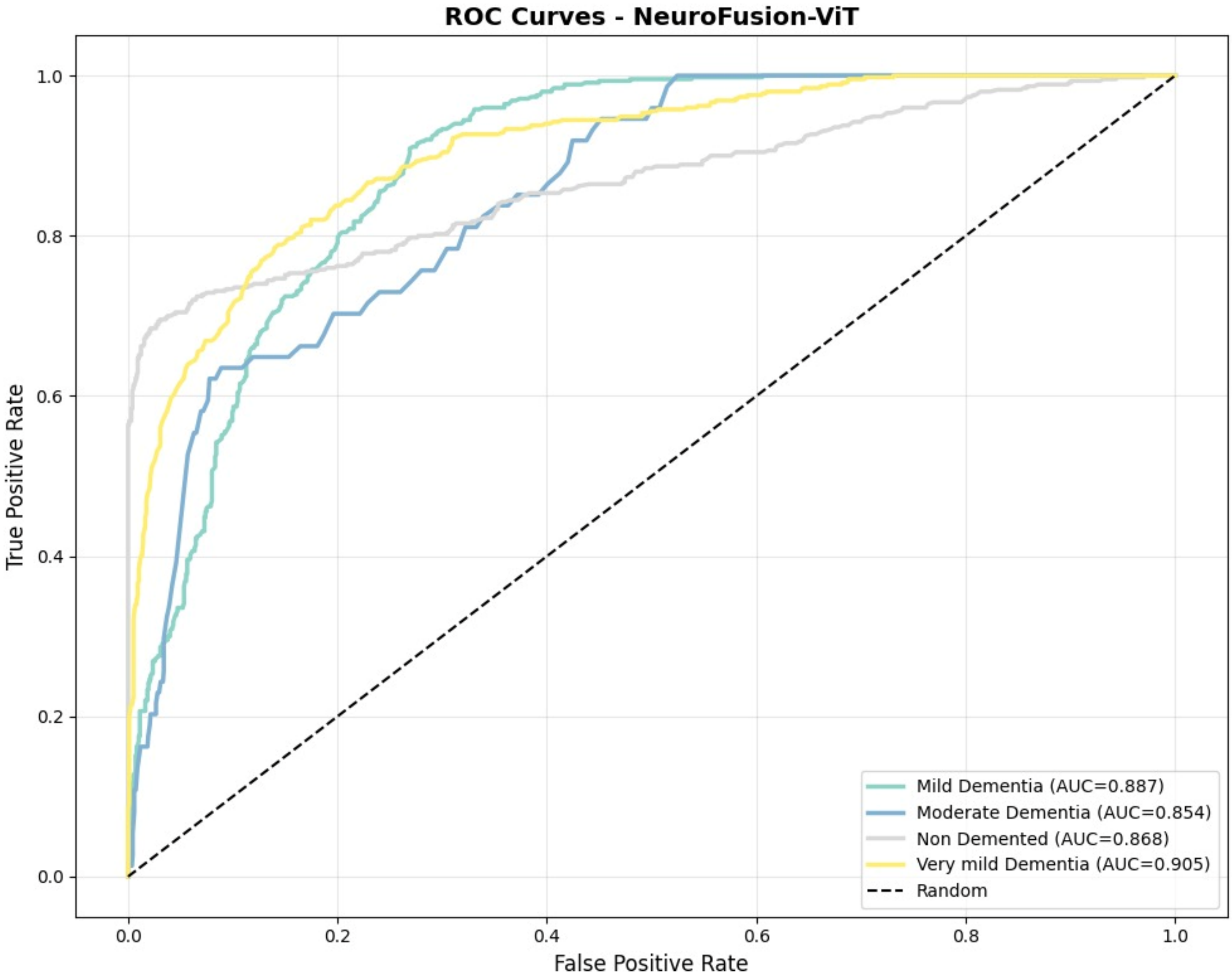

Figure S1. ROC curves for experiments E1–E4.
